# Absence of the Adaptor Protein PEA-15 Is Associated with Altered Pattern of Th Cytokines Production by Activated CD4+ T Lymphocytes *In Vitro*, and Defective Red Blood Cell Alloimmune Response *In Vivo*

**DOI:** 10.1371/journal.pone.0136885

**Published:** 2015-08-28

**Authors:** Stéphane Kerbrat, Benoit Vingert, Marie-Pierre Junier, Flavia Castellano, François Renault-Mihara, Silvina Dos Reis Tavares, Mathieu Surenaud, France Noizat-Pirenne, Jorge Boczkowski, Georges Guellaën, Hervé Chneiweiss, Sabine Le Gouvello

**Affiliations:** 1 Université Paris-Est, Créteil, France; 2 Inserm U955, Créteil, France; 3 Etablissement Français du Sang, Créteil, France; 4 Inserm, U1130, Neuroscience Paris Seine, IBPS, Paris, France; 5 Université Pierre et Marie Curie, UM119, Neuroscience Paris Seine, IBPS, Paris, France; 6 CNRS, UMR8246, Neuroscience Paris Seine, IBPS, Paris, France; 7 AP-HP, Hôpital H. Mondor- A. Chenevier, Pôle de Biologie-Pathologie, Créteil, France; Jackson Laboratory, UNITED STATES

## Abstract

TCR-dependent and costimulation signaling, cell division, and cytokine environment are major factors driving cytokines expression induced by CD4+ T cell activation. PEA-15 15 (Protein Enriched in Astrocyte / 15kDa) is an adaptor protein that regulates death receptor-induced apoptosis and proliferation signaling by binding to FADD and relocating ERK1/2 to the cytosol, respectively. By using *PEA-15*-deficient mice, we examined the role of PEA-15 in TCR-dependent cytokine production in CD4+ T cells. TCR-stimulated *PEA-15*-deficient CD4+ T cells exhibited defective progression through the cell cycle associated with impaired expression of cyclin E and phosphoRb, two ERK1/2-dependent proteins of the cell cycle. Accordingly, expression of the division cycle-dependent cytokines IL-2 and IFNγ, a Th1 cytokine, was reduced in stimulated *PEA-15*-deficient CD4+ T cells. This was associated with abnormal subcellular compartmentalization of activated ERK1/2 in *PEA-15*-deficient T cells. Furthermore, *in vitro* TCR-dependent differentiation of naive CD4^+^ CD62L+ *PEA-15*-deficient T cells was associated with a lower production of the Th2 cytokine, IL-4, whereas expression of the Th17-associated molecule IL4I1 was enhanced. Finally, a defective humoral response was shown in *PEA-15*-deficient mice in a model of red blood cell alloimmunization performed with Poly IC, a classical adjuvant of Th1 response *in vivo*. Collectively, our data suggest that PEA-15 contributes to the specification of the cytokine pattern of activated Th cells, thus highlighting a potential new target to interfere with T cell functional polarization and subsequent immune response.

## Introduction

The mechanisms by which naïve CD4+ T cells differentiate into different cell functional fates that include the T helper 1 (Th1), Th2, Th17, and T regulatory (Treg) cell lineages, characterized by a specific pattern of cytokines production, are of much interest in understanding how host immune response become adapted to different challenges [1,2]. Although cytokine environment and costimulation are major factors influencing CD4+ T cell differentiation, the cell division cycle is an intrinsic cue that has been involved in the specification of cytokine expression in Th cell, considering that DNA replication allows chromatin remodeling and demethylation of effector Th cytokine loci [3–6]. However, other studies suggested that DNA replication might not be mechanistically linked to Th cytokine specification [7,8]. Alternatively, different signaling pathways from the TCR complex itself were shown to influence the set of cytokine genes transcribed [9]. In particular, the involvement of ERK1/2 in T cell functional polarization was previously shown in different reports where the authors explain that TCR signal strength-dependent magnitude of ERK1/2 activity, as well as the duration of ERK1/2 activation control Th1 versus Th2 outcome of T cell activation [10–15], or Th17 versus regulatory T cell (Treg) differentiation [16–19]. ERK1/2 signaling has been also involved in regulation of G1 cell cycle progression [20]. However, whether ERK1/2 activation or compartmentalization contributes to cell cycle-dependent Th differentiation is not known.

The DED (death effector domain)-containing adaptor PEA-15 (Protein Enriched in Astrocytes/ 15k kDa) [21] regulates multiple cellular functions depending on the cell type. PEA-15 contains a nuclear export sequence that mediates the relocation of ERK1/2 from the nucleus to the cytoplasm, thereby regulating the Ras-dependent signaling pathway [22–25]. Another role of PEA-15 is to inhibit apoptosis induced by Fas, TNF-α or TRAIL, by interacting with the DED of FADD and caspase-8, and blocking DISC formation. The mitochondrial protein Htra2/OMI promotes apoptosis by targeting PEA-15 to proteasome (reviewed in [23]).

Here, we have taken advantage of *PEA-15*-deficient mice [26], to study the impact of PEA-15 deficiency on TCR-dependent specification of cytokine expression by CD4+ helper T cells.

## Materials and Methods

### Mice

PEA-15-deficient mice were previously described [26]. Mice were bred in our facility and backcrossed to a C57BL/6J background for 3 generations. For each experiment, PEA-15^-/-^ animals and PEA-15^+/+^ sex-matched littermates from PEA-15^+/-^ intercrosses, were used at 6–9 months of age. On the day of experiment, mice were sacrificed by cervical dislocation. All animals were maintained in our conventional animal facilities and manipulated in accordance with protocols approved by the Paris Est Creteil University (UPEC) ethical committee (COMETH, Authorization N°11/12/12-16), according to European guidelines.

### Flow cytometry and cell sorting

Spleen, lymph nodes, or thymus were harvested into a tissue culture dish and teased apart into a single cell suspension by pressing with the plunger of a 3 ml-syringe; the obtained cell suspension was passed through a cell strainer (20μm) to eliminate clumps and small fragments. Post-centrifugation supernatant was discard, and cell pellet resuspended in red blood cell lysing buffer. After centrifugation, supernatant was discard and cell pellet resuspended in staining media. A cell count and viability analysis were then performed. Flow cytometry studies were performed using a FC500 or a CyAn flow cytometer (both FC from Beckman-Coulter, Villepinte, France). Data were analysed using CXP software (Beckman-Coulter,Villepinte, France) or FlowJo software (v10.0.6 Tree Star, Ashland, OR). Antibodies for flow cytometry experiments were obtained from Caltag (San Francisco, CA, USA), BD Pharmingen, (San Diego, CA, USA), Beckman Coulter (Villepinte, France) and Miltenyi Biotec (Bergisch Gladbach, Germany). CD3^+^-, CD4^+^- or naïve CD4^+^CD62L^+^ T cells were enriched out of thymus or pooled lymph nodes (LN) and/or spleen cells suspensions as indicated, with negative selection magnetic cell sorting kit: pan T cell isolation kit mouse, CD4+ T cell isolation kit II mouse, CD4^+^CD62L^+^ T cell isolation kit II, respectively (Miltenyi Biotec, Bergisch Gladbach, Germany).

### Cell cycle and proliferation analysis

Cell cycle analysis of LN T cells was performed using Coulter Reagents Kit (Beckman-Coulter, Villepinte,France) on the basis of DNA staining with propidium iodide (PI) as described [27]. At least 30000 events were analyzed at low speed (100 events/second) and collected on list mode files. The percentage of T cells in the different phases of the cell cycle (i.e. G0/G1, S, G2/M phase) was determined. T lymphocyte proliferation capacity was analyzed after 24, 48 and 72H of culture of T cells with plate bound anti-CD3- (0.1/-1-2 μg/ml, as indicated in figure legend) with or without recombinant anti-CD28 (2μg/ml), with or without recombinant IL-2 (50U/mL) by enumeration in a haemocytometer after dilution of T cells in trypan blue, or by cytometric analysis of stimulated LN T cells labeled with the fluorescent dye carboxyfluorescein diacetate succinimidylester (CFSE) (CFDASE, Sigma-Aldrich, St Quentin Fallavier, France). Propidium iodide staining [1μg/ml] was performed in CFSE labelled cells [27]. In each histogram, the percentage of the dividing cells per cell generation was determined by quantification of CSFE cellular fluorescence halving using a flow cytometer CyAn (Beckman-Coulter, Villepinte, France), and data were analyzed using FlowJo software (v10.0.6 Tree Star, Ashland, OR).

### Cytokine production and IL4I1 activity analysis

The amount of IL2 in 18H culture supernatant of T cells stimulated with ConA, a mitogenic lectin that mimic anti-CD3 stimulation, was quantified by a CTLL2 bioassay, and the amount of IL-4 and IFNγ was quantified by Immunoassay (Quantikine, R&D systems Inc., Minneapolis, MN, USA).The amount of IFNγ, IL4, IL10 and IL17A produced in 5-days culture supernatant of plate bound anti-CD3- (0.1–1 μg/ml) stimulated naïve CD4^+^CD62L^+^ T cells with or without recombinant anti-CD28 (2μg/ml) was quantified by Luminex assay (MCYTOMAG-70K, Merck Millipore, Saint-Quentin en Yvelines, France). IL4I activity was quantified as already described by us [28].

### Western blot

NP-40 total cell lysates (50μg/line) of stimulated T cells were resolved on SDS-PAGE (6% for pRb-ppRb analysis; 12% for ERK1/2 & Akt analysis; 15% for cyclin E analysis) and transferred to 0.45μm pore size Immobilon-P PVDF membrane (Millipore, Billerica, MA, USA). Ab specific for phospho-ERK1/2, ERK1/2, Akt, phospho-Akt (Cell signaling technologies, Danvers, MA, USA), cyclin E (Santa Cruz, Dallas, TX, USA), or pRb (BD Biosciences, San Diego, CA) and actin (Sigma-Aldrich, St Quentin Fallavier, France) were used for immunoblotting, and immunoreactive bands were detected using ECF Western Blotting Reagent Pack (GE HealthCare Biosciences, Piscataway,NJ, USA), and analysed using a Storm Phosphorimager and the Image Quant software (GE HealthCare Biosciences, Piscataway,NJ, USA).

### Immunofluorescence

Stimulated purified LN T were spread on Superfrost plus slides (Menzel-Glaser, Braunshweig, Germany) at 1x10^5^ cells/slide after washing with PBS containing a cocktail of protease inhibitors: okadaic acid (1nM), NaF (10mM), sodium orthovanadate (1mM), and then fixed and permeabilized by PBS containing 4% formaldehyde for 15 minutes at room temperature. For anti-ERK1/2 staining, non-specific sites were blocked with goat serum (5%) (BioWest, Nuaillé, France) in PBS Triton X-100 (0.3%), incubated overnight at 4°C with anti-ERK1 (1:500, Santa Cruz, Dallas, TX, USA) in PBS-Triton X-100 (0,3%) and BSA.(0,1%), and subsequently for 1H at room temperature with Cy3 conjugated goat anti-rabbit antibody (1:1000, BD Biosciences, San Diego, CA). For anti-phospho ERK1/2 staining, cells were incubated in Triton X-100 (0.3%) for 30 minutes at room temperature before being fixed again with 50% methanol for 15 minutes at 4°C. After non-specific sites blocking, cells were incubated for 48H at 4°C with anti-phospho ERK1/2 (1:25, Cell Signaling technologies, Danvers, MA, USA) in PBS containing-Triton X-100 (0.3%) and 0,1% BSA, and then for 1H at room temperature with Alexa 488 conjugated goat anti-rabbit (1:5000, Life Technologies, Saint Aubin, France). A cocktail of phosphatase inhibitors was added in medium at each step of the procedure. After both staining, cells were washed with RNase A (1:20 in PBS, Life Technologies, Saint Aubin, France) for 1 minute, incubated with the DNA marker TOPRO3 (1:1000 in PBS, Life Technologies, Saint Aubin, France) for 20 minutes at room temperature, washed once with PBS and once with PB before being mounted in fluoromount-G medium (Southern Biotech, Birmingham, AL, USA) and analyzed on a Zeiss LSM 510 confocal microscope (Carl Zeiss, Oberkochen, Germany).

### RNA isolation and real time quantitative RT-PCR

Total mRNA isolation and qRT-PCR analysis were performed as published [29]. The sequences of primers are indicated in S1 Table. The expression of all the indicated target transcripts was measured by the relative quantification of real-time PCR using a mix of each cDNA sample as a calibrator sample, according to the ΔΔCt method [30].

### Measurement of anti-HEL IgG and Blood transfusion

Anti-HEL IgG flow-cytometry detection was based by flow cytometry crossmatch, and performed as described [31,32]. HEL-expressing HOD RBCs were incubated with a 1/10 dilution of serum sampling, followed by incubation with anti-mouse IgG labeled with APC (BD Biosciences, San Diego, CA). FVB RBCs were used a negative control. Anti-HEL antiserum was used as a positive control. Donor blood was leukoreduced with a neonatal leukoreduction filter (Purecell, Pall Biomedical, East Hills, NY). A total of 100 μl of leukoreduced RBC was injected via tail vein of recipient mice. Four hours before transfusion, recipients were injected intraperitoneally with either 100 μg of poly(I:C) (Pfizer, New York, NY) in 500 μl of PBS or with PBS alone. For Treg depletion, anti-CD25–purified mAb (clone PC61, rat IgG1, BIOXCELL West Lebanon, USA) were administered once intraperitoneally (500μg/mouse) 3 days before the transfusion as previously described [33,34]. The complete depletion of CD3^+^CD4^+^CD25^+^Foxp3^+^ T cells was checked by flow cytometry before blood transfusion. CD3-eF780-APC, CD4-PE-Cy7, CD25-APC, CD45RA-FITC mAbs used for Treg phenotyping were from BD Biosciences (San Diego, CA) and eBioscience (San Diego, CA). Cells were treated with a fixation/ permeabilization kit (eBioscience, San Diego, CA) and labeled with Foxp3-PE (eBioscience, San Diego, CA). All data were collected on an eight-color Canto II flow cytometer (BD, San Diego, CA) and analyzed using with FlowJo software (v10.0.6 Tree Star, Ashland, OR).

### Statistical analysis

All analyses were performed with Prism 5.0c software (GraphPad Software, La Jolla, CA, USA). Differences were considered significant if p < 0.05.

## Results

### PEA-15-deficient mice have impaired TCR-dependent T cell proliferation and Th1/2 cytokines production

**Fig 1:**
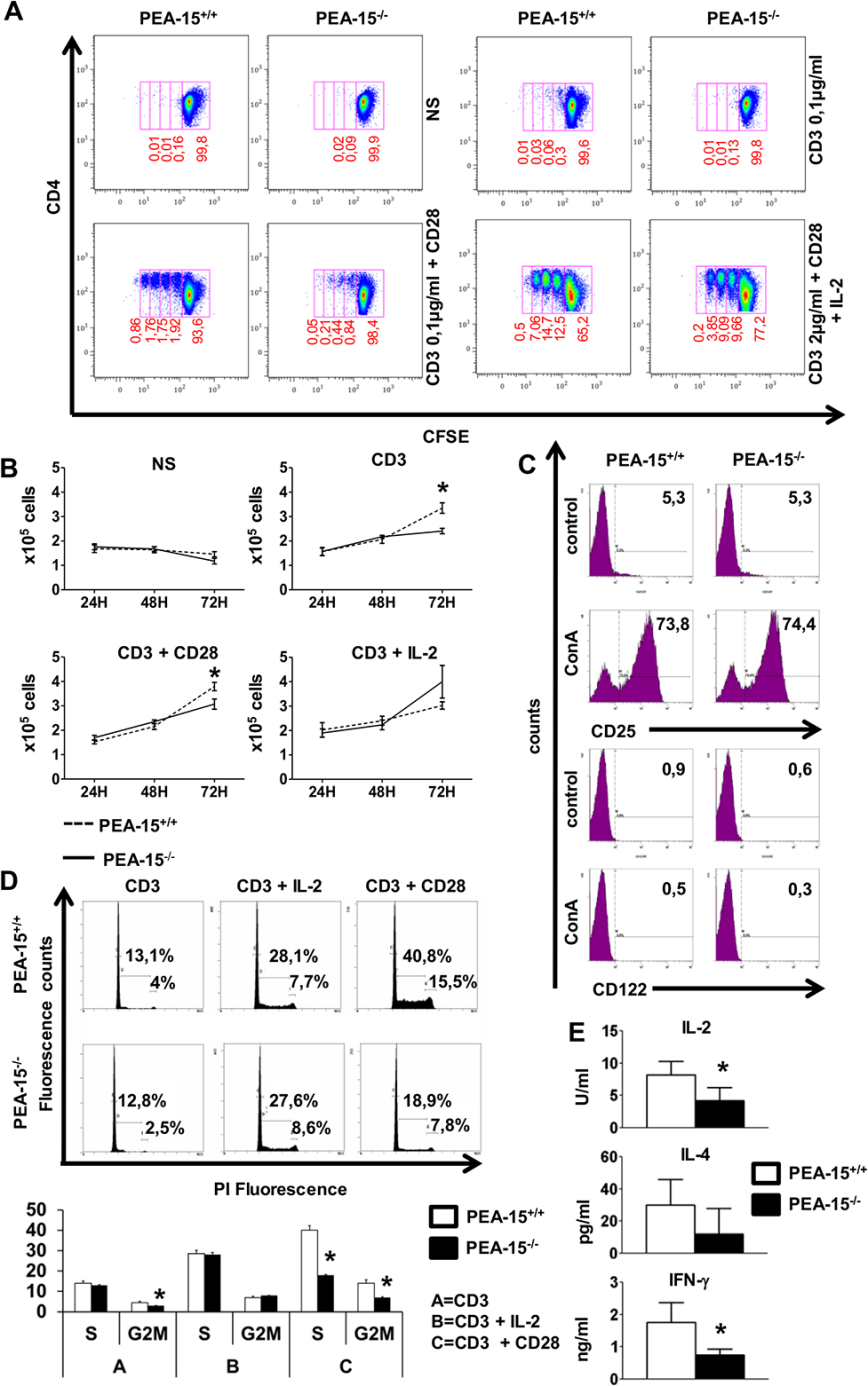
Activated PEA-15^-/-^ T cells have impaired proliferation, and reduced IL-2 & IFNγ production. **(A & B)** Purified lymph node (LN) CD4^+^ T cells of *PEA-15* ^-/—^and *PEA-15* ^+/+^ mice were stimulated with 0.1μg/ml or 2μg/ml (when indicated) of anti-CD3, with or without anti-CD28 (2μg/ml), in the presence or not of recombinant IL-2 (50U/mL). Proliferation was analyzed **(A)** by flow cytometry analysis of CFSE-labeled cells (numbers indicate the percentage of cells in each division at 72H, data are representative of 4 independent experiments**)** and **(B)** by enumeration of cells at the indicated time by trypan blue exclusion test. Data shown are means +/- SEM of 3 independent experiments (* p< 0.*05*). **(C)** *PEA-15* ^-/—^and *PEA-15* ^+/+^ T cells treated without (control) or with ConA mitogen for 18H, were analysed for CD25 and CD122 expression by flow cytometry at 12H and 24H after stimulation, respectively. The data shown were obtained using PE-labeled mAbs specific for CD25 and CD122 and FITC-labeled mAbs specific for CD4, and are gated on CD4-FITC positive cells. The percentage of cells in each quadrant is indicated. These values are representative of 6 experiments. **(D)** LN T cells from *PEA-15* ^-/-^ mice and *PEA-15* ^+/+^ littermates were treated as in (A & B) for 72H. Flow cytometry of cells stained with propidium iodide (PI) gated on CD4+ cells was performed for cell cycle analysis. Histograms of the percentage of cells in the S and G2/M phases of the cell cycle of a representative experiment (**D/**upper panel) and cumulative data of 4 independents experiments +/-SD (* p< 0.*05*) (**D/**lower panel) are shown. **(E)** Purified T cells from *PEA-15* ^-/-^ mice and *PEA-15* ^+/+^ littermates were stimulated as in (C). IL2 production in the culture supernatant was quantified by CTLL2 bioassay (n = 8; 3 independent experiments). Quantification of IL-4(Th2) and IFNγ (Th1) (n = 6; 3 independent experiments**)** in the culture supernatant was determined by ELISA (*p<0.*05)*.

We first tested the capacity of *PEA-15*-deficient T cells to proliferate and to produce IL-2, the T cell growth factor, as well as IFNγ and IL-4, the major Th1 and Th2 cytokines involved in CD4+-dependent cellular or humoral immune response, respectively. *PEA-15*-deficient lymph nodes (LN) T cells were incubated with low concentrations (0.1–2 μg/ml) of anti-CD3 mAbs, with or without anti-CD28 mAbs *in vitro*. Compared to *PEA-15*-proficient (wt) control T cells, *PEA-15*-deficient T cells proliferated less as demonstrated by slower kinetics of proliferating CFSE labeled *PEA-15* ^-/-^ CD4+ T cells (Fig 1; panel A) as well as reduced total cell number (Fig 1; panel B). Moreover, *PEA-15* ^-/-^ CD4+ T cells exhibited a reduced frequency of cells in the S phase upon incubation with anti-CD3 and anti-CD28 mAbs, and a reduced frequency of cells in the G2/M phase upon incubation with anti-CD3 mAb alone and together with anti-CD28 mAb (Fig 1; panel C). Accordingly, compared to their wild type (wt) littermates, lymph nodes of *PEA-15*-deficient mice contained a lower frequency of CD4^+^ T cells expressing a central memory CD62L^high^CD44^high^ phenotype which are homeostatically proliferating in lymph nodes (Table 1), whereas percentages of non proliferating effector/memory CD62L^low^CD44^high^ CD4^+^- and CD8^+^ LN T cells were similar in both murine lines. The percentage of naïve CD62L^high^CD44^low^ CD4^**+**^ **-** and CD8^**+**^ T cells were also similar in both murine lines, in accordance with the absence of alteration of thymus architecture or cellularity in *PEA-15*-deficient mice compared to wt mice (Table 1). In contrast, number of peripheral CD4^+^ and CD8^+^ splenocytes was lower in *PEA-15*-deficient mice compared to wt mice (Table 1). Addition of exogenous IL-2 suppressed the proliferation differences between *PEA-15*-deficient and-proficient T cells (Fig 1; panel A,-B,-C). Accordingly, activated *PEA-15*-deficient peripheral T cells secreted lower amounts of IL-2 than wt T cells (Fig 1; panel E). IFNγ and IL-4 production by activated *PEA-15*-deficient T cells was also reduced compared to wt T cells, although the difference for IL-4 production did not reach significance (Fig 1; panel E). Modulation of the expression level of the early activation marker CD25 which encode for the α chain of the IL-2 receptor, was similar at the surface of stimulated *PEA-15*-deficient- compared to wt T cells. Similarly, expression of CD122 which encode for the β chain of the IL-2 receptor, was similar at the surface of *PEA-15*-deficient- compared to wt T cells (Fig 1; panel D). Considering that PI labeling of CFSE positive cells showed no survival defect and neither higher susceptibility to AICD of *PEA-15*-deficient T cells (data not shown) as previously described [24], our results suggested that absence of PEA-15 is associated with defective cell cycling of CD4^+^ T cell and T cells reduced production of Th1/2 cytokines, both phenomenons potentially contributing to impaired proliferation capacity of PEA-15 ^-/-^ T cells.

**Table 1:** Lymphocytes composition of spleen, lymph nodes, and thymus in *PEA-15* ^-/—^mice. The cellular composition of spleen, lymph nodes, and thymus for *PEA-15* ^-/-^ mice and their WT littermates was determined by cell counting and/or flow cytometry after staining with mAbs specific for lymphocyte subsets. (N = 6; **: P< 0.05).

	**Spleen**	
	**PEA-15** ^**+/+**^	**PEA-15** ^**-/-**^
	Number of total splenocytes x 10^6^	
B cells	40,4 ± 18.2	24.8 ± 5.1
CD4+8- T cells	19.9 ± 1.8	16 ± 5.1 **
CD4-8+ T cells	12.6 ± 2.9	8.9 ± 1.5 **
	**Lymph nodes**	
	**PEA-15** ^**+/+**^	**PEA-15** ^**-/-**^
	% among total leukocytes	
B cells	16.4 ± 3.5	14.6 ± 4.5
CD4+8- T cells	43.5 ± 11	40.8 ± 7.9
CD4-8+ T cells	23.4 ± 3.5	22.9 ± 6
	% among CD4+ T cells	
CD62L^high^CD44^low^	75.8 ± 4.2	75.3 ± 7.4
CD62L^high^CD44^high^	5.7 ± 2.5	3.2 ± 1.1 **
CD62L^low^CD44^high^	10.4 ± 1.9	9.3 ± 3.5
	% among CD8+ T cells	
CD62L^high^CD44^low^	88.0 ± 2.1	87.8 ± 2.1
CD62L^high^CD44^high^	5.9 ± 1.6	4.6 ± 1.3
CD62L^low^CD44^high^	2.4 ± 0.7	2.9 ± 0.8
	**Thymus**	
	**PEA-15** ^**+/+**^	**PEA-15** ^**-/-**^
	% among total thymocytes	
CD4+ 8-	9.4 ± 0.5	11.7 ± 1.2
CD4+ 8+	76.3 ± 2.3	68.1 ± 2.4
CD4- 8-	11.4 ± 3.1	16.5 ± 3
CD4- 8+	2.9 ± 0.6	3.6 ± 0.6

### PEA-15 regulates subcellular compartmentalization and activity of phosphoERK

**Fig 2:**
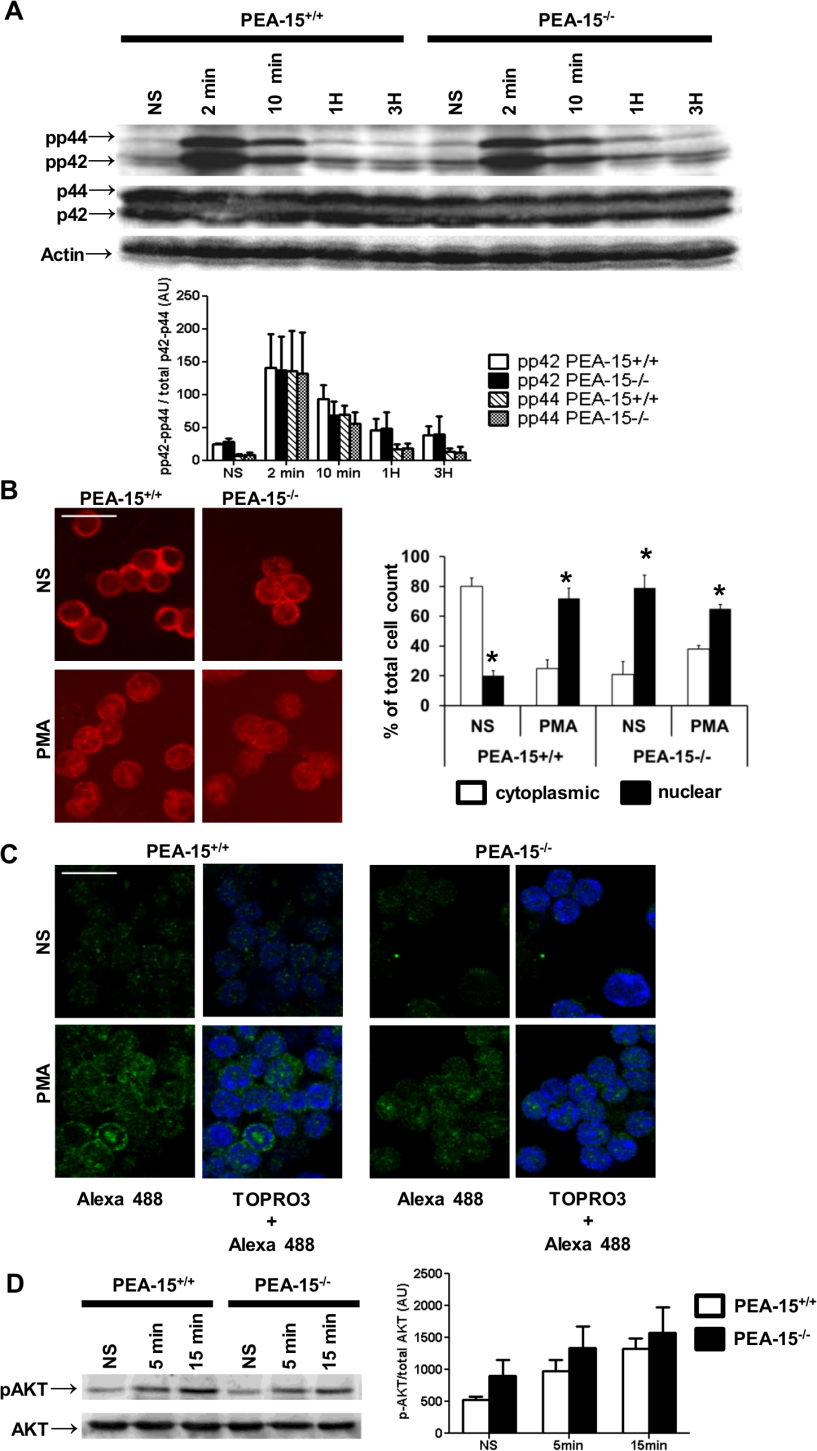
PEA-15 regulates ERK1/2 and phosphoERK1/2 subcellular compartmentalization. (A) Negatively sorted *PEA-15* ^+/+^ or *PEA-15* ^-/-^ CD4^+^ T lymphocytes were stimulated with cross-linked anti-CD3 mAbs (1μg/ml) for the indicated times. Total cell lysates (50μg/line) were resolved by SDS-PAGE followed by western-blotting. Total ERK-1/2 was detected by the mean of anti-p42/p44 antibodies; ERK-1/2 phosphorylation was assessed with anti-phospho-ERK1/2 antibodies. Quantitative densitometric analysis of phospho-p42 and phospho-p44 expression out of 4 experiments is presented below a representative immunoblot. Results are expressed as means +/-SEM (n = 4). (B & C) ERK1/2 localization was assessed by immunofluorescence in resting cells (NS) or cells stimulated with PMA (200nM) for 15 minutes, using an anti-P42/P44 antibody. A representative experiment is shown in (B); histograms represent the % of enumerated cells in which the ERK1/2 staining was cytoplasmic and/or nuclear. Values are means +/- SD of 4 independent experiments (* *p<0*.*05*). (C) Phospho-ERK1/2 localization was assessed in the same stimulation conditions as in (B) using an anti-phospho ERK-1/2 antibody. Nuclei were stained with TOPRO3. (D) Expression of phospho-Akt and Akt was assessed in sorted CD4+ T lymphocytes from *PEA-15* ^+/+^ or *PEA-15* ^-/-^ mice stimulated or not with cross-linked anti-CD28 mAb (5μg/ml) for 5 or 15 min. 50μg of total cell lysate proteins were resolved by 12% SDS-PAGE followed by western-blotting. Representative results out of 4 independent experiments are shown. Histograms representing densitometric analysis of phospho-Akt expression are shown on the right of the panel. Results are expressed as means +/- SEM of 4 independent experiments.

PEA-15 regulates ERK1/2 compartmentalization in different cell types [22–25]. Proper cell cycling and activation of ERK1/2 are linked [20]. Thus we investigated whether defective CD4+ T cell cycling in *PEA-15*-deficient mice was associated with abnormal activation/compartmentalization of ERK1/2. After stimulation with phorbol 12-myristate 13-acetate (PMA) which bypass the membranous steps of TCR/CD3 complex-dependent stimulation of ERK1/2, T cells from *PEA-15*-deficient and wt mice expressed similar expression levels of total ERK1/2 (p42/p44)- as well as phosphorylated ERK1/2 (pp42/pp44) (Fig 2; panel A). However, whereas incubation with PMA induced the translocation of part of total ERK1/2 from the cytoplasm to the nucleus in wt T cells, our results show that total ERK1/2 was already mainly localized in the nucleus in resting *PEA-15*-deficient T cells (Fig 2; panel B), confirming that PEA-15 contributed to maintain location of some ERK1/2 in the cytoplasm of T lymphocytes [24]. Moreover, phosphoERK1/2 was also mainly detected in the nucleus in activated *PEA-15*-deficient T cells, whereas it was both nuclear and cytoplasmic in activated wt T cells (Fig 2; panel C). We then tested PI3K-signaling pathway downstream CD28 triggering, which might modulate TCR-signaling either via the ERK1/2 pathway or via the PI3K pathway [35,36]. Our results showed that CD28 activation of the PI3K-dependent phosphorylation of Akt did not differ between *PEA-15*-deficient T cells as compared to control wt cells (Fig 2; panel D).

**Fig 3:**
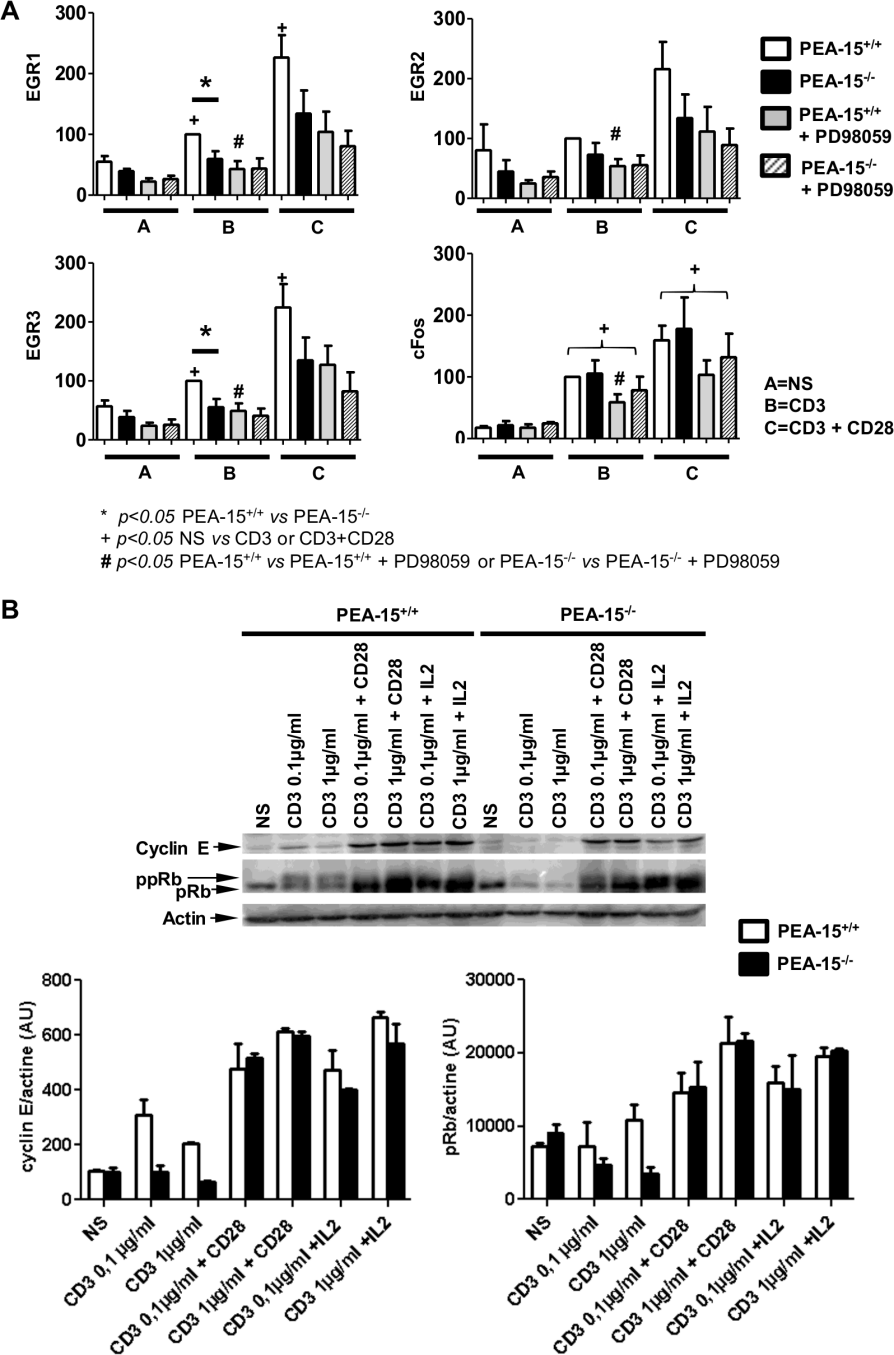
Dysregulation of ERK signaling dependent—targets in TCR-stimulated PEA-15^-/-^ T cells. (A) Negatively sorted CD4+ T lymphocytes from *PEA-15* ^-/-^ mice and *PEA-15* ^+/+^ littermates were preincubated or not with the MEK/ERK inhibitor PD98059 (30μM) for 30 minutes, and then stimulated with cross-linked anti-CD3 mAbs (0,1μg/ml), with or without anti-CD28 mAbs (2μg/ml) for 30 min. Indicated genes expression was quantified by real-time quantitative PCR. Means +/- SEM from 5 separate experiments are shown, and expressed as percentage of the “CD3 (0.1μg/ml)-stimulated-*PEA-15* ^+/+^-CD4^+^-T-cells” condition taken as positive control. Statistical significance is indicated *p<0.*05*: for comparison between PEA-15^+/+^ or PEA-15^-/-^; + *p<0*.*05*: for pairwise comparison of different culture condition groups; *# p<0*.*05*: between cells treated without or with PD98059; (Mann-Whitney *U* test). (B) Negatively sorted CD4+ T lymphocytes from *PEA-15* ^-/-^ mice and *PEA-15* ^+/+^ littermates were stimulated for 30 min with anti-CD3 (0.1–1μg/ml), with or without anti-CD28 (5μg/ml) mAbs, or with or without rIL-2 (100U/ml). Whole cell lysates were prepared, and equal amounts of proteins were resolved by SDS-PAGE and blotted with anti-cyclin E mAbs, or with anti-pRb mAbs. A representative experiment out of 3 is shown. Arbitrary densitometric units for the bands were analysed by computing densitometry. Means +/-SEM (n = 3) for pRb and cyclin E expression are represented below the immunoblot.

Then we investigated the expression level of the immediate early growth response genes (EGR)-1, -2, -3 and cFos, which are classical transcriptional targets of the ERK1/2 signaling pathway [37]. Basal expression level of these four genes was similar between *PEA-15*-proficient and–deficient CD4+ T cells. Stimulation with anti-CD3 mAb induced a higher expression of EGR-1 and EGR-3 and to a lesser extent of EGR-2 in wt T cells, while it did not in *PEA-15*-deficient T cells (Fig 3; panel A). Addition of anti-CD28 mAbs enforced the anti-CD3-stimulated expression of EGR-1, -2 and -3 in *PEA-15*-proficient T cells. In *PEA-15*-deficient T cells, simultaneous addition of anti-CD3- and anti-CD28 mAbs stimulated the expression of EGR-1, -2, -3 although it did not reach significant level compared to resting cells (EGR1: p = 0.0635; EGR2: p = 0.063; EGR3: p = 0.19). To confirm the involvement of ERK1/2 signaling in modulation of EGR1,-2,-3 expression in *PEA-15* proficient T cells, we treated wt cells with the MEK inhibitor (PD98059) before stimulation. As expected, PD98059 prevented the CD3- and CD3+CD28 –stimulated enhancement of EGR-1, -2, -3 expression level in wt T cells; in accordance with defective PEA-15-dependent regulation of ERK1/2 activity, PD98059 treatment of stimulated *PEA-15*-deficient T cells had no effect on EGR-1, -2, -3 expression. Similarly, although expression of cFos was similarly up-regulated in *PEA*-15-deficient T cells and control wt cells after stimulation with anti-CD3 mAb in addition or not with anti-CD28 mAb; PD98059 treatment reduced this up-regulated expression only in the stimulated the wt line (Fig 3; panel A).

Considering that proper cyclin E expression and pRb phosphorylation by ERK1/2 in the nucleus is required for progression into the S phase [20], we then investigated the effect of *PEA-15* deficiency on pRb and cyclin E regulation. pRb and cyclin E were expressed at the same weak level in *PEA-15*-deficient T cells and wt control cells in the resting state. Incubation with anti-CD3 mAb alone induced pRb phosphorylation in wt cells, while it did not in *PEA-15*-deficient T cells (Fig 3; panel B). Upon incubation with anti-CD3 mAb and either with anti-CD28 mAb or rIL-2, pRb was phosphorylated in both wt and *PEA-15*-deficient T cells. Similar results were obtained when using 0.1μg/ml or 1 μg/ml of anti-CD3 mAb. Likewise, upon incubation with anti-CD3 mAb, cyclin E expression was induced in wt- but not in *PEA-15*-deficient T cells (Fig 3; panel B). Costimulation with anti-CD28 mAb or addition of exogenous rIL-2 enhanced the CD3-stimulated expression of cyclin E in wt cells and in *PEA-15*-deficient T cells. Altogether, our data suggest that abnormal localization of phosphoERK1/2 in *PEA-15*-deficient T cells is associated with defective regulation of some downstream targets of the ERK1/2 signaling pathway.

### PEA-15 modulates the set of cytokines expressed upon TCR-stimulation of naive CD4+ T cells

**Fig 4:**
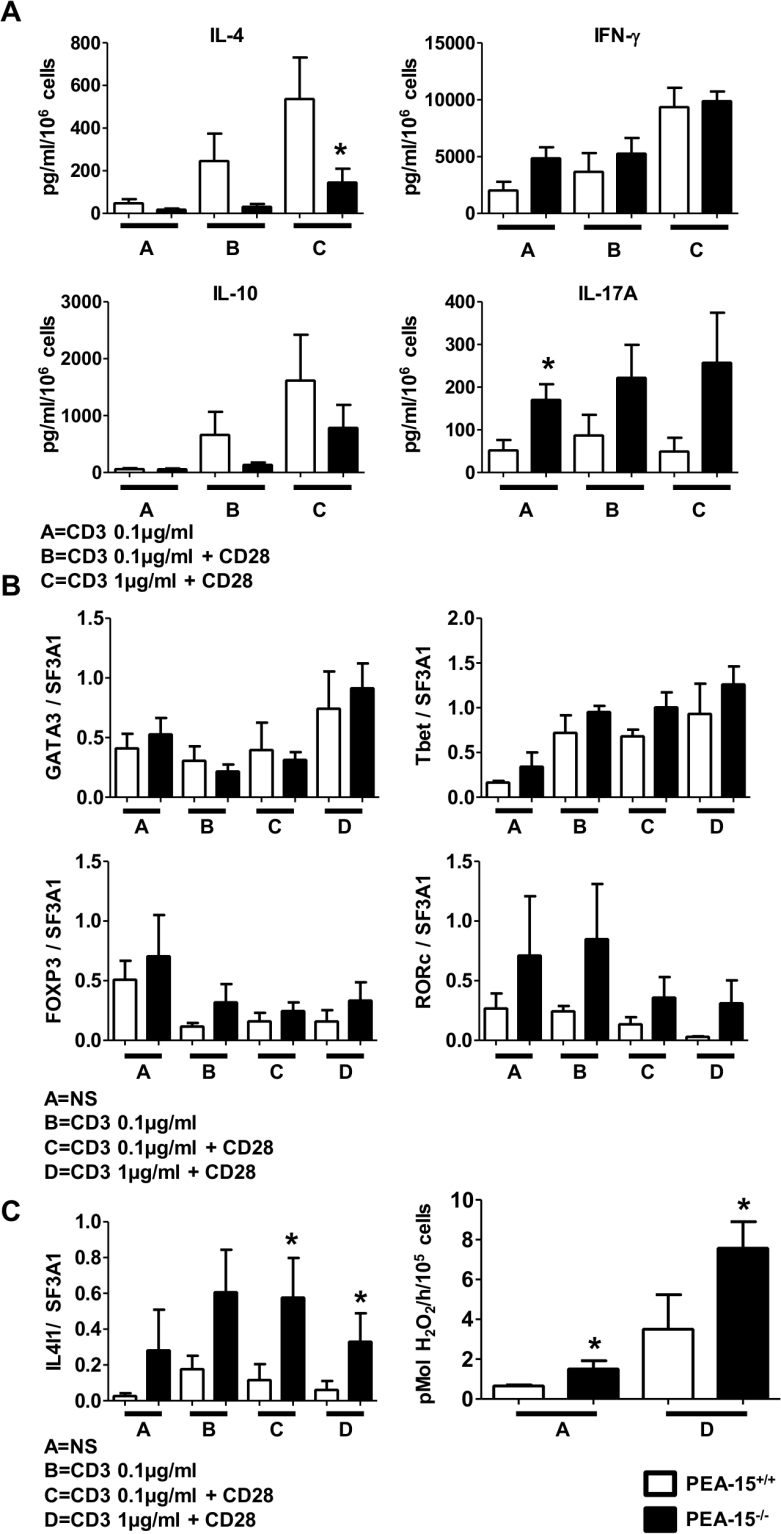
Activated PEA-15^-/-^ T cells have decreased IL-4 production, and increased activity of IL4I1. (A, B & C) Sorted naïve CD4^+^ CD62L^+^ T lymphocytes from *PEA-15* ^-/-^ mice (■) and *PEA-15* ^+/+^ (□) mice were stimulated with anti-CD3 (0.1–1 μg/ml) with or without anti-CD28 (2μg/ml), for 5 days. (A) Cytokines production was quantified in the culture supernatants by Luminex assay. (B) GATA3, Tbet, FOXP3 and RORc, and IL4I1 (C left panel) genes expression was analyzed by real-time quantitative PCR in total mRNA extracts of the cultures. Means +/- SEM from n = 6 out of 2 independent experiments are presented. (C right panel) IL4I1 activity was measured in 10^5^ CD4^+^ CD62L^+^ T lymphocytes stimulated or not with plate-bound anti-CD3*-* (1 μg/ml) and soluble anti-CD28 (2μg/ml) mAbs for 5 days. Means +/- SEM from four separate experiments are presented. Statistical significance is indicated for comparison between *PEA-15* ^+/+^- and *PEA-15* ^-/-^ T cells, *p<0.05 (Mann-Whitney U test).

**Fig 5:**
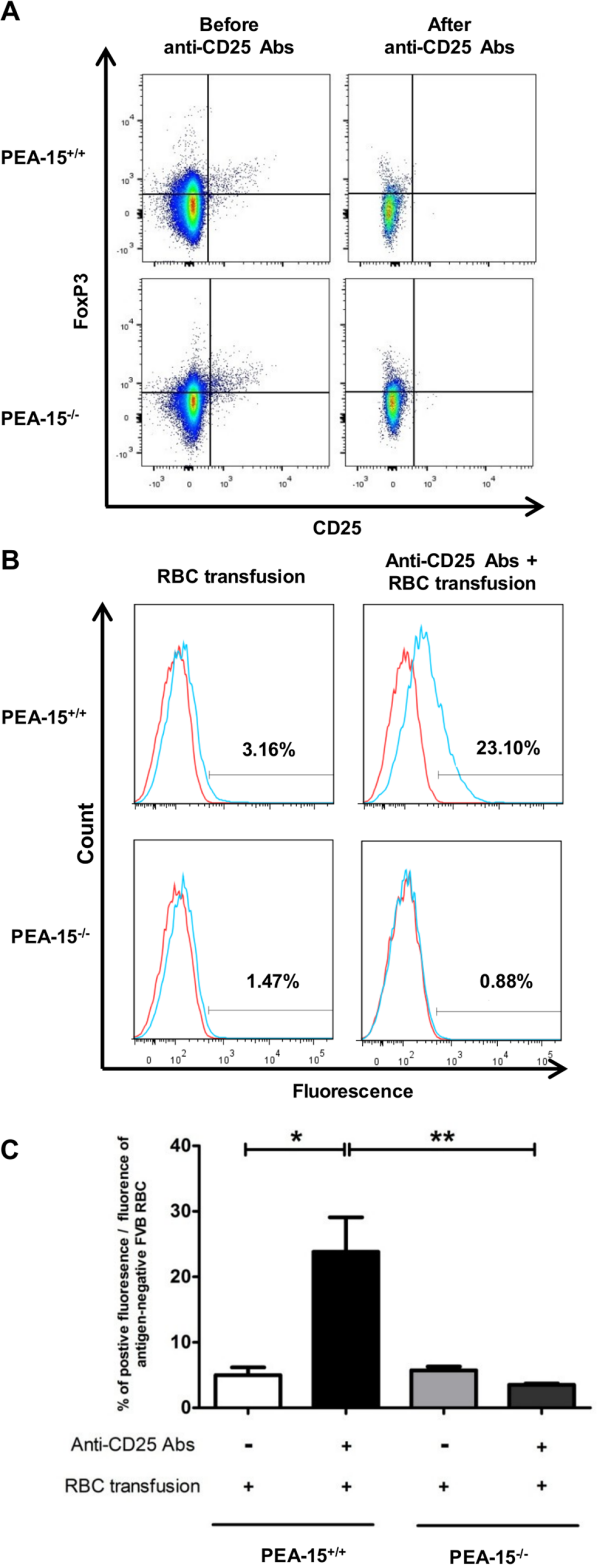
Resistance of PEA-15^-/-^ mice to HEL+RBC alloimmunization. Three days before the red blood cell (RBC) transfusion, *PEA-15* ^-/—^or *PEA-15* ^+/+^ mice were depleted in Treg with an anti-CD25 mAbs. At day 0, mice were transfused with a leukoreduced blood from HEL^+^ HOD mice, 4 hours after injection of the adjuvant poly (I:C),. (A) Representative Dot plots showing % of CD25+ Foxp3+ Treg among CD4+ T-cells before and after *in vivo* Treg depletion with anti-CD25 Abs; (B) Histograms showing representative data of anti-HEL IgG in sera from *PEA-15* ^-/—^or *PEA-15* ^+/+^ mice using flow cytometry-based mHEL crossmatch. HEL^+^ HOD RBCs (blue line) or HEK-negative FVB RBCs (red line; negative control) were incubated with a 1/10 dilution of sera from transfused *PEA-15* ^-/-^ or *PEA-15* ^+/+^ mice (n = 10; 2 independent experiments). (C) For each group of mice, the % of the positive mean fluorescence intensity was defined as the mean fluorescence of the serum crossmatched with HEL-negative FVB RBCs substracted from the mean fluorescence of the serum crossmatched with HEL^+^ HOD RBCs. Statistical analysis were performed with the Mann-Whitney test. * p<0.05; **p<0.01.

We then investigated how absence of *PEA-15* would impact the specification of cytokine production by naïve CD62L^high^ CD4^+^ T cell stimulated by anti-CD3 and anti-CD28 mAbs, non-polarizing Th0 condition of functional differentiation in the absence of exogenously added polarizing cytokines). Compared to wt cells, *PEA-15*-deficient T cells secreted less IL-4 and exhibited a trend to secrete less IL-10 (Fig 4; panel A). Conversely, CD3-stimulated *PEA-15*-deficient T cells secreted more IL-17 compared to stimulated wt cells. IFN-γ expression levels were similar between the lines in the different stimulation conditions. We then measured the expression of mRNA coding for the transcription factors T-bet, GATA-3, RORc and FoxP3 that are master regulators of Th1, Th2, Th17 and T_reg_ cells differentiation, respectively [2]. While *PEA-15*-deficient and wt T cells exhibited similar levels of T-bet, GATA-3 and FoxP3, RORc seemed to be expressed at higher levels in *PEA-15*-deficient T cells although this difference did not reach statistical significance (Fig 4; panel B). Furthermore, compared to wt cells, *PEA*-15-deficient T cells expressed (Fig 4; left panel C) and secreted more IL-4-induced gene 1 (IL4I1) (Fig 4; right panel C), a phenylalanine oxidase [28,38,39], whose mRNA expression was recently showed in Th17 to be strictly dependent on RORc expression [38]. We next investigated whether the lower production of IL-4 and IL-10 by TCR-stimulated *PEA-15*-deficient CD4^+^ CD62L+ naïve T cells *in vitro*, was associated with an abnormal humoral immune response *in vivo*. To this aim, we used the previously described red blood cells (RBC) alloimmunization model in which mice are injected intraperitoneally with poly(I:C) and then transfused with Hen Egg Lysozyme (HEL)-conjugated RBC [31,32]. In order to sensitize the model by preventing potential suppression, prior injection with anti-CD25 mAbs was performed before transfusion, to deplete Treg before alloimmunization (Fig 5; panel A) [33,34]. Mice were sacrificed after one week, and serum levels of anti-HEL IgG were measured by cross-matched flow cytometry. While wt mice exhibited readily detectable anti-HEL IgG in their serum, *PEA-15*-deficient mice did not, demonstrating that PEA-15 was necessary for antibody production in this model (Fig 5; panel B & panel C).

## Discussion

In this paper, we have found that *PEA-15* deficiency in CD4+ T cells resulted in constrained T cell cycling and impaired production of IL-2 and IFNγ by activated mature CD4+ T cells *in vitro*, as well as impaired production of IL-4 and to a lesser extent IL-10 by TCR-stimulated differentiating CD4+CD62L+ naïve T cells. Conversely, higher expression and activity of IL4I1, a Th17-associated phenylalanine oxidase was induced in TCR-stimulated *PEA-15*-deficient CD4^+^ CD62L+ naïve T cells. These abnormalities were associated with defective humoral response to RBC alloimmunization in *PEA-15*-deficient mice *in vivo*. In parallel, our results showed that absence of *PEA-15* resulted in abnormal subcellular compartmentalization of phosphoERK1/2 in resting and activated CD4+ T cells, and this was associated with impaired regulation of classical targets of the ERK1/2 signaling pathway. Although indirectly, our data suggest that PEA-15-dependent regulation of cytokines expression in CD4+ T cells, involves lower ERK1/2-signaling, in accordance with other reports, showing that subcellular compartmentalization of ERK1/2 represents another level of regulation of the enzymes activity, besides phosphorylation of ERK1/2 [40–42]. The resident nuclear ERK1/2 in resting *PEA-15*-deficient T cells, confirms the data reported by Pastorino et al. [24]; it may be the result of previous *in vivo* ERK1/2 activation followed by dephosphorylation by nuclear phosphatases and defect of return of ERK1/2 to the cytoplasm due to *PEA-15* deficiency, both mechanisms resulting in lower amount of the enzyme available in the cytoplasm for a subsequent activation. In agreement with this hypothesis, crystal structure analysis recently illustrated that PEA-15 binding triggers an extended allosteric conduit in dually phosphorylated ERK2, disrupting key features of active ERK2 and at the same time PEA-15 binding protects ERK2 from dephosphorylation and finally prepares it to be released at a given place for a given target [25]. In support of the regulatory role of PEA-15 on amplitude of ERK1/2 activity, is the lower expression of the ERK1/2 transcriptional target *EGR1* [37] in stimulated *PEA-15*-deficient T cells compared to stimulated *PEA-15*-proficient T cells; conversely, ERK1/2 signal duration seems not to be not affected by PEA-15 absence, as suggested by the similar *c-Fos* expression level shown in both lines [43]. Pretreatment of CD3-stimulated *PEA-15*-deficient T cell with the MEK/ERK inhibitor (PD98059) had no effect on *EGR1*,*-2*,*-3* expression compared to the inhibitory effect of this pretreatment on expression of the same four genes in CD3-stimulated wt T cells; this further supports the involvement of ERK1/2 in impaired *EGRs* expression shown in *PEA-15*-deficient T cells. Moreover, the lower expression of *EGR1* in stimulated *PEA-15*-deficient T cells may contribute to reduced IL-2 expression [44,45] and lower counts of proliferating *PEA-15*-deficient T cells due to impaired IL-2-dependent ERK autocrine loop [18,46]. Further, the lower expression of *EGR1* may also contribute to reduced IL-4 expression in stimulated-naïve *PEA-15*-deficient T cells [47]. This would be in accordance with reports showing that Th2 differentiation was positively regulated by ERK [12–15]. Impaired IL-2 and/or IL-4 secretion by TCR/CD28- stimulated *PEA-15*-deficient cells could also contribute to the higher expression of IL-17A and IL4-I1 shown in mutant cells compared to *PEA-15*-proficient cells, in accordance with previous results showing that IL-2 constrains Th17 cell generation and that IL-4 negatively regulates T helper cell production of IL-17A [48–50]. However, direct effect of altered ERK1/2 activity on enhanced expression of Th17-related molecules in *PEA-15*-deficient T cells cannot be excluded, as MEK/ERK1/2 signaling was shown to regulate Th17 differentiation, positively or negatively, depending on the pharmacologic inhibitor used [16,17].

In our model of RBC alloimmunization, we have used poly(I:C), a classical adjuvant for Th1 responses [51]. Thus the lower IL-2 and IFNγ production after activation of mature *PEA-15*-deficient CD4+ T cells compare to wt CD4+ T cells, could contribute to the defective humoral alloimmune response to RBC observed *in vivo* in Treg-depleted *PEA-15*-deficient mice. Moreover, impairment of IL-10 production could contribute to the abnormal humoral response to RBC observed *in vivo* in *PEA-15*-deficient mice. Indeed, IL-10 plays a major role in B cell differentiation and Ig switching [52,53]. However, our results do not allow to exclude the potential contribution of abnormal antigen presenting cells- and B cells proper functions due to *PEA-15* deficiency in these cells, in the defective humoral alloimmune response to RBC observed in our model.

Alternatively, another mechanism that could contribute to explain reduced IL-4, IL-10 and IFNγ production by stimulated *PEA-15*-deficient CD4+ T cells, could be the defective cell cycling [3–6] of TCR-stimulated *PEA-15* ^-/-^ T cells, associated with the reduced levels of cyclin E expression and phosphorylation of pRb, both molecules regulating the G1 to S/G2/M transition and being ERK-dependent [54]. Similar expression of GATA-3, the master regulator of IL-4 transcription, found in the mutant and control T cells might be explained by the cell cycle independence of GATA-3 expression [55]. Further, the impaired proliferation of *PEA-15*-deficient T cells when treated with both anti-CD3- and anti-CD28 mAbs might derive from a partial block in mitosis due to the ERK1/2-dependent arm of CD28-dependent signaling [56]. Conversely, the similar phosphorylation of Akt in PEA-15-deficient and–proficient T cells after stimulation with anti-CD28 mAb suggested that the PI3-kinase-dependent arm of CD28-dependent signaling [57,58] did not contribute to the defective proliferation of *PEA-15*-deficient T cells, in contrast with the effect of the other DD (death domain)-adaptor c-FLIP on T-cell activation, which was proposed to be PI3K-dependent [59]. Finally, a higher sensitivity of *PEA-15-*deficient CD4+ T cells to Fas-dependent AICD [60] cannot be evoked to explain the lower frequency of CD4+ T cells reported in *PEA-15*-deficient mice; indeed, in accordance with Pastorino et al. [24], we showed that Fas-dependent AICD was preserved in *PEA-15-*deficient T cells, in contrast to the anti-apoptotic function of PEA-15 in fibroblasts, gliomas and astrocytes [23]. Some of our results contrast with those reported by Pastorino *et al*. [24], who claimed that PEA-15 negatively regulated T cell proliferation and IL-2 production. This discrepancy could be due to the much greater amount of anti-CD3 mAb, and therefore the strength of TCR signaling [10,11,15] used in Pastorino et al.’s experiments [24], which could account for the higher anti-TNP Ig levels due to higher Th1/Th2 cytokines production. It is notable that we found that the PEA-15-deficient mice do not have spontaneous adenopathy, as similarly reported by Pastorino *et al*. [24] which already noticed that this observation was rather paradoxical compared to the PEA-15-dependent negative regulation of T cell proliferation suggested by their others results. However, the differences in immunization conditions, for example the subcutaneous route of immunization used in this latter work, as well as the difference in the genetic background, could also explain the discrepancy of this result compared to ours. Altogether our findings demonstrate that PEA-15 contributes to the specification of the cytokine repertoire downstream the TCR-dependent activation of naïve T cells which contributes to regulation of Th differentiation. Although indirectly, our results suggest also that this phenomenon is dependent on PEA-15-dependent ERK1/2 activity regulation. Thus, it might be speculated that the PEA-15/ERK2 axis is an attractive target for therapeutic approaches aiming at suppressing the T cell-dependent activation and differentiation.

## Supporting Information

S1 Table Sequences of oligonucleotide primers used for quantitative RT-PCR studies. (PDF)
