# Supplementary material for: Absence of the Adaptor Protein PEA-15 Is Associated with Altered Pattern of Th Cytokines Production by Activated CD4+ T Lymphocytes In Vitro, and Defective Red Blood Cell Alloimmune Response In Vivo
Source: PLoS One. 2015 Aug 28;10(8):e0136885. doi: 10.1371/journal.pone.0136885 (PMC4552951; doi:10.1371/journal.pone.0136885)
Supplement: S1 Table — (PDF) [file pone.0136885.s001.pdf]

S1 Table . Sequences of oligonucleotide primers used for quantitative RT-PCR studies:

Gene name	GenBank Accession N°		Sequence 5'-3'	Product Size (bp)
**SF3A1**	NM_026175	Forward :	CCACTGAGTCCAAACAGCCAAT	161
		Reverse :	AGCTTCAAATTCAGGCCCAT	
**c-Fos**	NM_010234	Forward :	CAGAGCGGGAATGGTGAAGA	194
		Reverse :	GTTGATCTGTCTCCGCTTGGA	
**EGR1**	NM_007913	Forward :	CAATCTGTACCCCGAGGAGATC	63
		Reverse :	TCAGAGCGATGTCAGAAAAGGA	
**EGR2**	NM_010118	Forward :	CTCGCCGCCTCGTCGGT	174
		Reverse :	CTGGTTTCTAGGTGCAGAGATGGGA	
**EGR3**	NM_018781	Forward :	ACTCGGTAGCCCATTACAATCAG	180
		Reverse :	TGTCCTGGCACCAGTTGGA	
**Foxp3**	NM_054039	Forward :	TCCACAACATGCGACCCCCTTTCA	217
		Reverse :	ACAGCCCCCTTCTCGCTCTCCA	
**GATA3**	NM_008091	Forward :	GCCCCTCATTAAGCCCAAGCGA	169
		Reverse :	CAGGGGTCTGTTAATATTGTGAAGCTTGTAGT	
**IL4I1**	NM_010215	Forward :	GCTGGGCTACAACCTGAACAAC	202
		Reverse :	CACATCTCCCAGGAGCTGCA	
**RORC**	NM_011281	Forward :	TCTGCAAGTCCTTCCGAGAGA	175
		Reverse :	GCAAACTCCACCACATACTGAATG	
**T-bet**	NM_019507	Forward :	ATCGTGGAGGTGAATGATGGAGAGC	288
		Reverse :	GCTGGGAACAGGATACTGGTTGGATAGA	
